# Characterization of whole mitogenome sequence of the Tongde yak (*Bos grunniens*)

**DOI:** 10.1080/23802359.2021.1958082

**Published:** 2021-07-27

**Authors:** Guangzhen Li, Xudong Wei, Shengmei Chen, Ruizhe Li, Sangjia Zhou, Wenhao Li, Yuan Lin, Mohammed Yosri, QuratulAin Hanif, Zhijie Ma

**Affiliations:** a Academy of Animal Science and Veterinary Medicine, Qinghai University, Xining, China; b Key Laboratory of Plateau Livestock Genetic Resources Protection and Innovative Utilization, Xining, China; c Agriculture, Animal husbandry and Water Conservancy Bureau of Tongde County in Qinghai Province, Tongde, China; d The Regional center for Mycology and Biotechnology, Al Azhar University, Cairo, Egypt; e Animal Genomics Lab, Agricultural Biotechnology Division, National Institute for Biotechnology and Genetic Engineering, Faisalabad, Pakistan

**Keywords:** *Bos gru*nniens, genetic characterization, mitogenome, annotation

## Abstract

Tongde County is located in the southeast of Qinghai Province, China, harboring rich yak genetic resources. In the present study, the complete mitochondrial genome (mitogenome) of the Tongde yak (*Bos grunniens*) was firstly sequenced using Illumina sequencing technique and the corresponding sequence characterization was identified. Our results showed that the mitogenome of Tongde yak is a circular molecule with 16,323 bp length consisting of 37 genes (13 protein-coding genes, 2 rRNA genes, 22 tRNA genes) and a non-coding control region (D-loop), which is consistent with most bovine species. The overall nucleotide composition was found as: A (33.72%), T (27.27%), C (25.80%), and G (13.21%), respectively, yielding a higher AT content (60.99%). The complete mitogenome sequence of Tongde yak would provide useful information for further studies on its genetic resource conservation and molecular breeding programmes in the future.

Yak (*Bos grunniens*) is a valuable *Bovine* species, endemic to the Qinghai-Tibetan Plateau (QTP). In China, there are 20 officially recognized yak breeds and ∼15 million yak heads are inhabitant in this high-elevation ecosystem (National Committee of animal genetic resources 2021). Qinghai Province, located in the northwest of China and northeast region of QTP, has abundant yak genetic resources with more than five million individuals. Due to special geographical location, plateau climate and long history of yak breeding, some exceptional yak breeds/populations have been formed and identified recently in Qinghai province. For instance, two developed breeds (Datong and Ashidan) and four indigenous breeds (Gaoyuan, Huanhu, Xueduo and Yushu) harbor in this environment (National Committee of animal genetic resources 2021). Based on the archaeological analyses, mitochondrial and Y chromosomal variations, Qinghai is believed to be the center of origins and/or domestication for the yak (Wiener et al. 2003; Guo et al. 2006; Ma 2019). Tongde yak (*Bos grunniens*), harboring a strong high-altitude adaptation and resistance characteristics with a population size of around 250,000 yak heads, is found in Tongde County of Qinghai Province. The mammalian mitochondrial genome (mitogenome) is characterized by tachytelic evolution, simple structure and easy sequencing. The genetic characteristics of the mitogenome are one of the most important means to explore the maternal origin, diversity and migration history of mammals (Ingman et al. 2000; Shapiro and Hofreiter 2014; Reynolds et al. 2020). At present, mitogenomes of wild yak and some domestic yak breeds or populations have been successively assembled and annotated (Wang et al. 2021). However, no information is available on the mitogenome of Tongde yak. Therefore, in the current study, we sequenced and assembled it’s mitogenome sequence, which would provide useful basic data for the molecular genetic evaluation of this population.

Here, blood sample of one Tongde yak (*Bos grunniens*) was collected in Tongde County, Qinghai, China (100°20′N, 35°3′E). The voucher specimen (TD-1-20200824) is stored in the Key Laboratory of Plateau Livestock Genetic Resources Protection and Innovative Utilization of Qinghai Province, Academy of Animal Science and Veterinary Medicine, Qinghai University (Xining, Qinghai Province, China). The genomic DNA was extracted using DNA Extraction Kit (Aidlab Biotechnologies Co., Ltd, China), and stored at −20 °C for later use. The complete genome of Tongde yak was sequenced using HiSeq 2000 platform (Illumina) with sequencing depth of 23.3×. The reads were aligned to the wild yak mitochondrial reference genome (Accession number: NC_006380) using the Burrows-Wheeler Alignerv0.7.15 (Li and Durbin 2009) with the sub-command < aln -t 24 − 11024 -n 0.01 -o 2>, which were subsequently converted to BAM files using the command samtools view -Sb. To improve alignment into the circularized genome, the 30 bp of sequence from the end of the mtDNA was attached to the beginning. InDel realignment was performed using the Genome Analysis ToolKit (GATK v3.8) (McKenna et al. 2010), determining the circular mitogenome. The accurate annotated mitogenome sequence of Tongde yak was submitted to GenBank with the accession number MZ313873. The length of mitogenome was 16,323 bp, consisting of 13 protein-coding genes, 22 tRNA genes, two rRNA genes and one non-coding region (D-loop region) (Table 1). The gene composition, structure and arrangement of mitogenome for Tongde yak are similar to most other bovine species (Pramod et al. 2018; Prabhu et al. 2019; Kamalakkannan et al. 2020; Wang et al. 2021). All mitochondrial genes of Tongde yak (*Bos grunniens*) are encoded on the heavy strand except for the eight tRNA and *ND6* genes. The mitogenome base contents as follows: A 33.72%; C 25.80%; G 13.21% and T 27.27%, which yielded a higher AT content (60.99%) than GC content (39.01%). The total length of the protein-coding gene sequences was 11,282 bp. The most protein-coding genes initiate with ATG except for three gene (*ND2*, *ND3* and *ND5*), which begin with ATA. Three overlapping sequences between protein-coding genes in the same strand were found: ATP8 overlapped with ATP6 for 34 bp, *ND4L* overlapped with *ND4* for 4 bp. The overlap of the *ATPase* genes appears to be common in most vertebrate mitochondrial genome (Clayton 2000). Eight protein-coding genes terminate with TAA whereas the *ND2* gene terminates with TAG while, *Cytb* gene terminates with AGA. Moreover, an incomplete stop codon (T- -) is used in *ND4*, *ND3* and *COX3*. Twenty-two tRNA genes were interspersed in the mitochondrial genome and ranging from 60 to 75 bp in length. The *12S* and *16S rRNA* genes were 957 bp and 1572 bp in length, respectively. The putative control region (D-loop), a 893 bp fragment, was located between the *tRNA^Pro^* and *tRNA^Phe^*, which is responsible for transcription and replication of the mitochondrial genome.

**Table 1.** Summarized characterization of whole mitogenome sequence of Tongde yak (*Bos grunniens*).

	Position			Base composition (%)						
				
Gene	From	To	Size (bp)	A	C	G	T	Start codon	Stop codon	Strand
D-loop	1	893	893	32.47	25.32	13.77	28.44			H
*tRNA^Phe^*	894	960	67	34.33	22.39	19.4	23.88			H
*12S rRNA*	961	1917	957	36.47	22.57	18.18	22.78			H
*tRNA^Val^*	1918	1984	67	38.81	19.4	11.94	29.85			H
*16S rRNA*	1983	3554	1572	38.13	20.75	17	24.12			H
*tRNA^Leu^*	3556	3630	75	32	22.67	17.33	28			H
*ND1*	3639	4583	945	32.43	28.87	12.24	26.46	ATG	TAA	H
*tRNA^Ile^*	4589	4657	69	40.58	10.14	15.94	33.33			H
*tRNA^Gln^*	4655	4726	72	36.11	27.78	9.72	26.39			L
*tRNA^Met^*	4729	4797	69	27.54	27.54	18.84	26.09			H
*ND2*	4798	5835	1037	37.24	27.26	8.06	27.45	ATA	TAG	H
*tRNA^Trp^*	5840	5906	67	37.31	20.9	16.42	25.37			H
*tRNA^Ala^*	5908	5976	69	39.13	23.19	10.14	27.54			L
*tRNA^Asn^*	5978	6051	74	31.08	28.38	14.86	25.56			L
OL	6052	6082	31	35.48	29.03	25.81	9.68			L
*tRNA^Cys^*	6084	6150	67	29.85	26.87	19.4	23.88			L
*tRNA^Tyr^*	6151	6218	68	29.41	20.59	16.18	33.82			L
*COX1*	6220	7758	1539	28.74	25.44	16.31	29.51	ATG	TAA	H
*tRNA^Ser^*	7762	7830	69	33.8	28.17	14.08	23.94			L
*tRNA^Asp^*	7838	7905	68	36.23	17.39	17.39	28.99			H
*COX2*	7907	8587	681	34.36	22.66	14.47	28.51	ATG	TAA	H
*tRNA^Lys^*	8594	8660	67	31.34	17.91	20.9	29.85			H
*ATP8*	8662	8856	195	41.79	22.89	5.97	29.35	ATG	TAA	H
*ATP6*	8823	9497	675	33.33	26.73	11.31	28.63	ATG	TAA	H
*COX3*	9503	10,285	783	26.12	29.45	15.24	29.19	ATG	T– –	H
*tRNA^Gly^*	10,287	10,355	69	31.88	20.29	15.94	31.88			H
*ND3*	10,356	10,700	345	30.92	28.9	12.14	28.03	ATA	T– –	H
*tRNA^Arg^*	10,703	10,771	69	39.13	10.14	11.59	39.13			H
*ND4L*	10,772	11,065	294	31.99	23.23	11.78	33	ATG	TAA	H
*ND4*	11,062	12,429	1368	33.38	27	10.01	29.61	ATG	T– –	H
*tRNA^His^*	12,440	12,509	70	41.43	15.71	8.57	34.29			H
*tRNA^Ser^*	12,510	12,569	60	31.67	18.33	16.67	33.33			H
*tRNA^Leu^*	12,571	12,640	70	37.14	15.71	8.57	34.29			H
*ND5*	12,647	14,443	1797	32.89	29	10.65	27.46	ATA	TAA	H
*ND6*	14,451	14,969	519	42.23	29.36	7.58	20.83	ATG	TAA	L
*tRNA^Glu^*	14,973	15,041	69	39.13	21.74	11.59	27.54			L
*Cytb*	15,046	16,186	1140	31.75	28.86	13.07	26.32	ATG	AGA	H
*tRNA^Thr^*	16,189	16,258	70	36.23	24.64	14.49	26.64			H
*tRNA^Pro^*	16,258	16,323	66	33.33	28.79	13.64	24.24			L

## Data Availability

The data that support the findings of this study are openly available in GenBank of NCBI at https://www.ncbi.nlm.nih.gov, accession number MZ313873.

## Sampling and uploading information of voucher personnel

Sample collection personnel include Guangzhen Li, Zhijie Ma, and Shengmei Chen, submitted the voucher is Guangzhen Li and Zhijie Ma. The Email of Guangzhen Li: guangzhenli6228@126.com; Zhijie Ma: zhijiema@126.com.
